# Automation of the kidney function prediction and classification through ultrasound-based kidney imaging using deep learning

**DOI:** 10.1038/s41746-019-0104-2

**Published:** 2019-04-26

**Authors:** Chin-Chi Kuo, Chun-Min Chang, Kuan-Ting Liu, Wei-Kai Lin, Hsiu-Yin Chiang, Chih-Wei Chung, Meng-Ru Ho, Pei-Ran Sun, Rong-Lin Yang, Kuan-Ta Chen

**Affiliations:** 1 Big Data Center, China Medical University Hospital, China Medical University, Taichung, Taiwan; 2 Kidney Institute and Division of Nephrology, Department of Internal Medicine, China Medical University Hospital and College of Medicine, China Medical University, Taichung, Taiwan; 3 Institute of Information Science, Academia Sinica, Taichung, Taiwan; 4 Information Office, China Medical University Hospital, China Medical University, Taichung, Taiwan

**Keywords:** Ultrasonography, Epidemiology, Outcomes research

## Abstract

Prediction of kidney function and chronic kidney disease (CKD) through kidney ultrasound imaging has long been considered desirable in clinical practice because of its safety, convenience, and affordability. However, this highly desirable approach is beyond the capability of human vision. We developed a deep learning approach for automatically determining the estimated glomerular filtration rate (eGFR) and CKD status. We exploited the transfer learning technique, integrating the powerful ResNet model pretrained on an ImageNet dataset in our neural network architecture, to predict kidney function based on 4,505 kidney ultrasound images labeled using eGFRs derived from serum creatinine concentrations. To further extract the information from ultrasound images, we leveraged kidney length annotations to remove the peripheral region of the kidneys and applied various data augmentation schemes to produce additional data with variations. Bootstrap aggregation was also applied to avoid overfitting and improve the model’s generalization. Moreover, the kidney function features obtained by our deep neural network were used to identify the CKD status defined by an eGFR of <60 ml/min/1.73 m^2^. A Pearson correlation coefficient of 0.741 indicated the strong relationship between artificial intelligence (AI)- and creatinine-based GFR estimations. Overall CKD status classification accuracy of our model was 85.6% —higher than that of experienced nephrologists (60.3%–80.1%). Our model is the first fundamental step toward realizing the potential of transforming kidney ultrasound imaging into an effective, real-time, distant screening tool. AI-GFR estimation offers the possibility of noninvasive assessment of kidney function, a key goal of AI-powered functional automation in clinical practice.

## Introduction

The main clinical application of kidney ultrasound imaging involves excluding reversible causes of acute kidney injury, such as urinary obstruction, or identifying irreversible chronic kidney disease (CKD) that precludes unnecessary workup such as kidney biopsy.^1^ Its noninvasiveness, low cost, lack of ionizing radiation, and wide availability make it an attractive option for frequent monitoring and follow-up of the longitudinal change in kidney length and sonographic characteristics of kidney cortex relevant to kidney functional change. However, the high subjective variability in image acquisition and interpretation makes it difficult to translate experience-based prediction into standardized practice, such as invasive serum creatinine measurement. Yet, noninvasive imaging techniques for organ functional and structural characterization have been increasingly investigated aiming to minimize the invasive approach in both diagnostic and screening settings. Lorenzo et al. has recently proposed a unique pediatric CKD care model balancing cost and minimizing invasiveness with the improvement of risk prediction by using kidney ultrasound imaging to predict the development of CKD and surgical outcomes in infants with hydronephrosis.^2^

Conventionally, nephrologists tend to use kidney length and volume and cortical thickness and echogenicity to evaluate the severity of kidney injury. Very short renal length (e.g., <8 cm), apparent white cortex, and contracted capsule contour, all indicate an irreversible kidney failing process with high specificity but limited sensitivity.^3^ Furthermore, whether these ultrasonographic parameters can be used to predict accurate estimated glomerular filtration rates (eGFRs) remains controversial. For instance, studies^3–14^ have reported that although kidney length is highly specific in detecting irreversible CKD, its correlation with eGFR was only weak to moderate, ranging no association to 0.66. Even if only studies using the conventional Modification of Diet in Renal Disease Study (MDRD) equation to estimate eGFR (MDRD-eGFR) are considered,^15^ the best correlation noted between kidney length and eGFR has been only 0.36.^8,16^ Similarly, a fair-to-moderate correlation of kidney volume and cortical echogenicity with eGFR has been reported.^6,11,14,17,18^ By contrast, cortical thickness seems to be better correlated with MDRD-eGFR than is kidney length, with a correlation coefficient as high as 0.85.^3,8–13,16,18^ However, the study reporting the correlation coefficient of 0.85 was obtained for a small sample of 42 adults with CKD without validation.^8^ Yapark et al.^5^ developed a CKD scoring system integrating three ultrasonographic parameters, namely kidney length, parenchymal thickness, and echogenicity, to improve the correlation; however, the correlation was moderate (*r* = 0.587).^5^ Furthermore, the assigned score of each parameter remains subjective with undefined interobserver reliability.^5,19^

To overcome substantial interobserver variability in kidney ultrasound interpretation, machine learning provides a solid and objective foundation for analytic standardization to inform clinical decisions. Recent advances in image segmentation, classification, and registration through deep learning have considerably expanded the scope and scale of medical image analysis.^20^ Deep learning-oriented diagnostic applications may minimize unnecessary and invasive procedures, thus greatly improving the efficiency and sustainability of current health care systems. Moreover, with the phenomenal increase in computing performance, real-time computer-aided diagnosis may further change mobile telecare and telemedicine. In the current CKD care model, it remains controversial whether kidney function should be routinely screened in all asymptomatic adults.^21^ The most commonly used CKD screening tests include testing the urine for protein or testing the blood for serum creatinine; however, there is no conclusive evidence suggesting which screening test is more appropriate to the other in the context of routine screening. Developing an easily available and noninvasive image marker of kidney function using deep learning methods thus may provide a valuable complimentary tool for diagnosing CKD. To explore this possibility in clinical practice, we developed a deep learning algorithm based on both kidney ultrasound imaging and clinical data in a large registry-based CKD cohort.

## Results

### Study population

The median age of 1299 included patients was 65 years; 582 (45%) of them were men. Moreover, 41% and 74.7% were diagnosed as having diabetes and hypertension, respectively (Table 1). The median serum creatinine level and eGFR were 2.07 (interquartile range [IQR]: 1.40–4.29) mg/dl and 30.12 (IQR: 12.56–48.72) ml/min/1.73 m^2^, respectively. The distribution of eGFR is summarized in Supplemental Fig. 1.

**Table 1:** The clinical characteristics of the study datasets at patient and image level

Variables	Patients (*N* = 1297)	Ultrasound Images (*N* = 4505)	Ultrasound Images in nontesting dataset (*N* = 4,010)	Ultrasound Images in testing dataset (*N* = 495)	*p* value^a^
Demographics, median (IQR)					
Age (years)	65 (53, 74)	65 (52, 74)	65 (53, 74)	63 (51, 74)	0.269
Male, n (%)	715 (55.1)	2464 (54.7)	2201 (54.9)	263 (53.1)	0.459
Comorbidity, n (%)					
Cardiovascular disease	1001 (77.2)	3504 (77.8)	3117 (77.7)	387 (78.2)	0.820
Hypertension	968 (74.6)	3405 (75.6)	3024 (75.4)	381 (77.0)	0.446
Diabetes	533 (41.1)	1888 (41.9)	1697 (42.3)	191 (38.6)	0.112
Biochemical value, median (IQR)					
Serum creatinine (mg/dL)	2.1 (1.4, 4.4)	2.1 (1.4, 4.3)	2.1 (1.4, 4.4)	2.0 (1.4, 3.6)	0.003
eGFR (mL/min/1.73 m^2^)	30.0 (12.3, 48.6)	30.1 (12.6, 48.7)	29.9 (12.3, 48.2)	31.6 (15.7, 55.5)	0.001
Sonographic parameter, median (IQR)					
Kidney length (cm)	9.72 (8.76, 10.53)	9.62 (8.69, 10.51)	9.63 (8.66, 10.51)	9.54 (8.80, 10.48)	0.703

^a^*p* values denotes probability for difference between the nontesting and testing datasets and are calculated by Wilcoxon rank sum test for continuous variables and Chi-square test (or Fisher’s exact test as appropriate) for categorical variables

### Predicting eGFR through convolutional neural networks

**Fig. 1:**
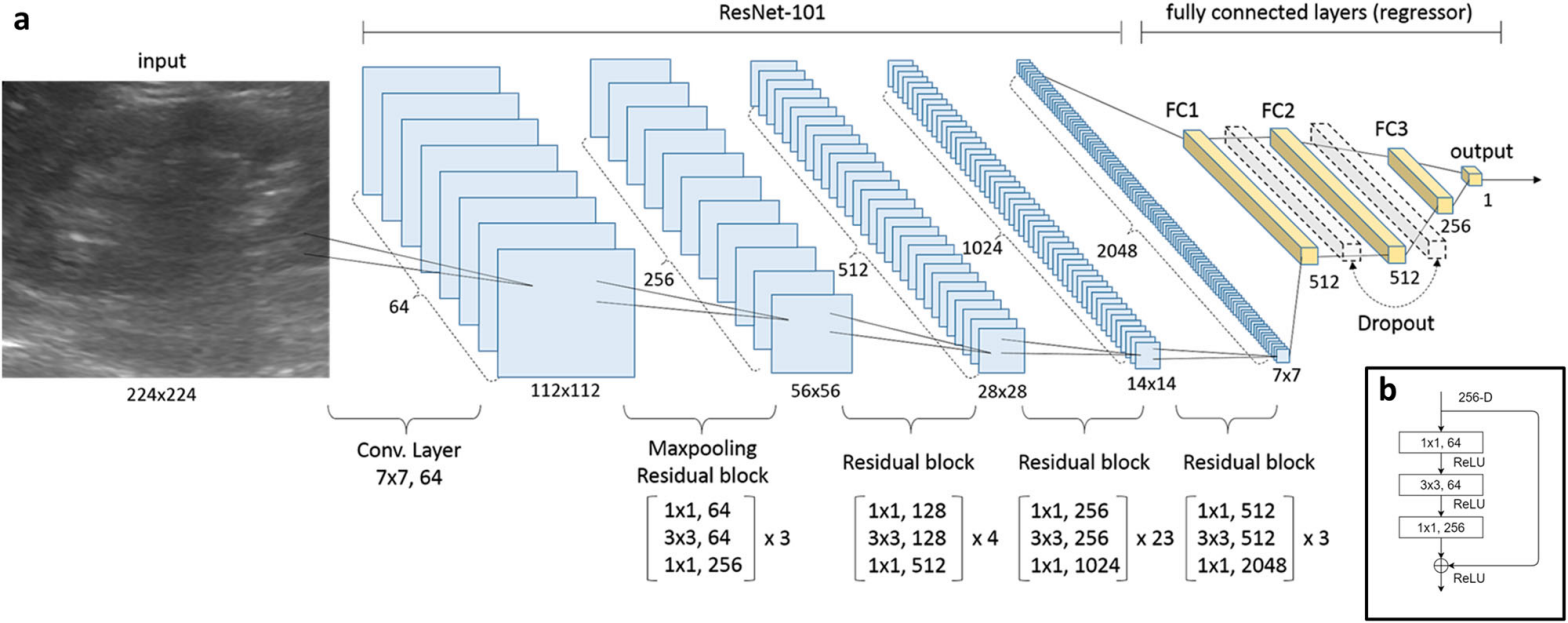
CNN architecture for kidney function estimation based on kidney sonographic images. **a** Proposed neural network architecture included 33 residual blocks (100 convolution layers in total) as a CNN-based feature extractor and three fully connected layers of 512, 512, and 256 neurons as a regressor for eGFR prediction. Feature maps are colored in blue and the regressor is specified in yellow. The dropout probability was set at 0.5. (**b**) Components of the first residual block in the CNN

**Fig. 2:**
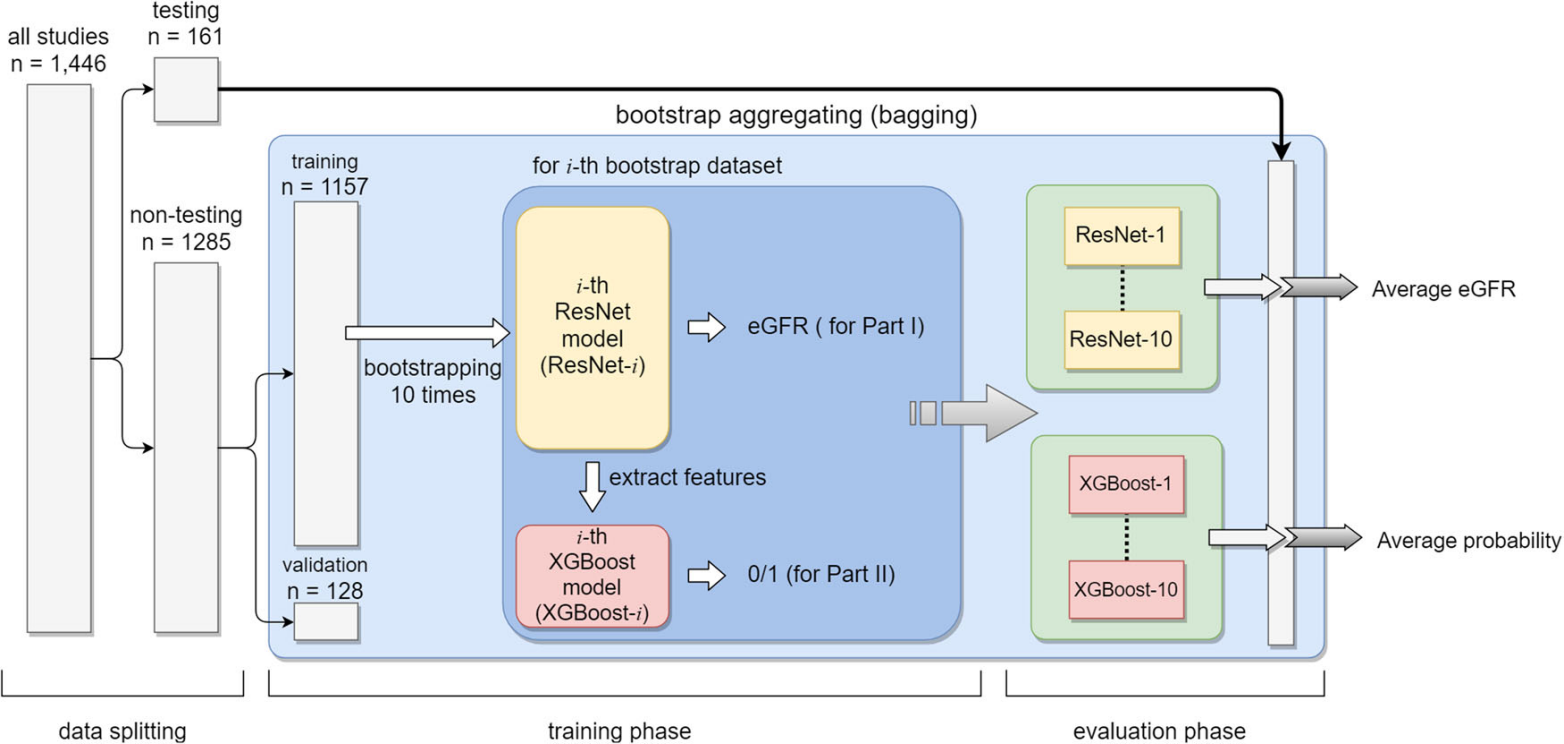
Flowchart shows the summary of the data processing from bagging in the training phase to the final evaluation phase. Briefly, we obtained 10 ResNet models for predicting continuous eGFR and 10 XGBoost models for CKD status classification. In the evaluation phase, we averaged the output of 10 models as the final prediction result

**Fig. 3:**
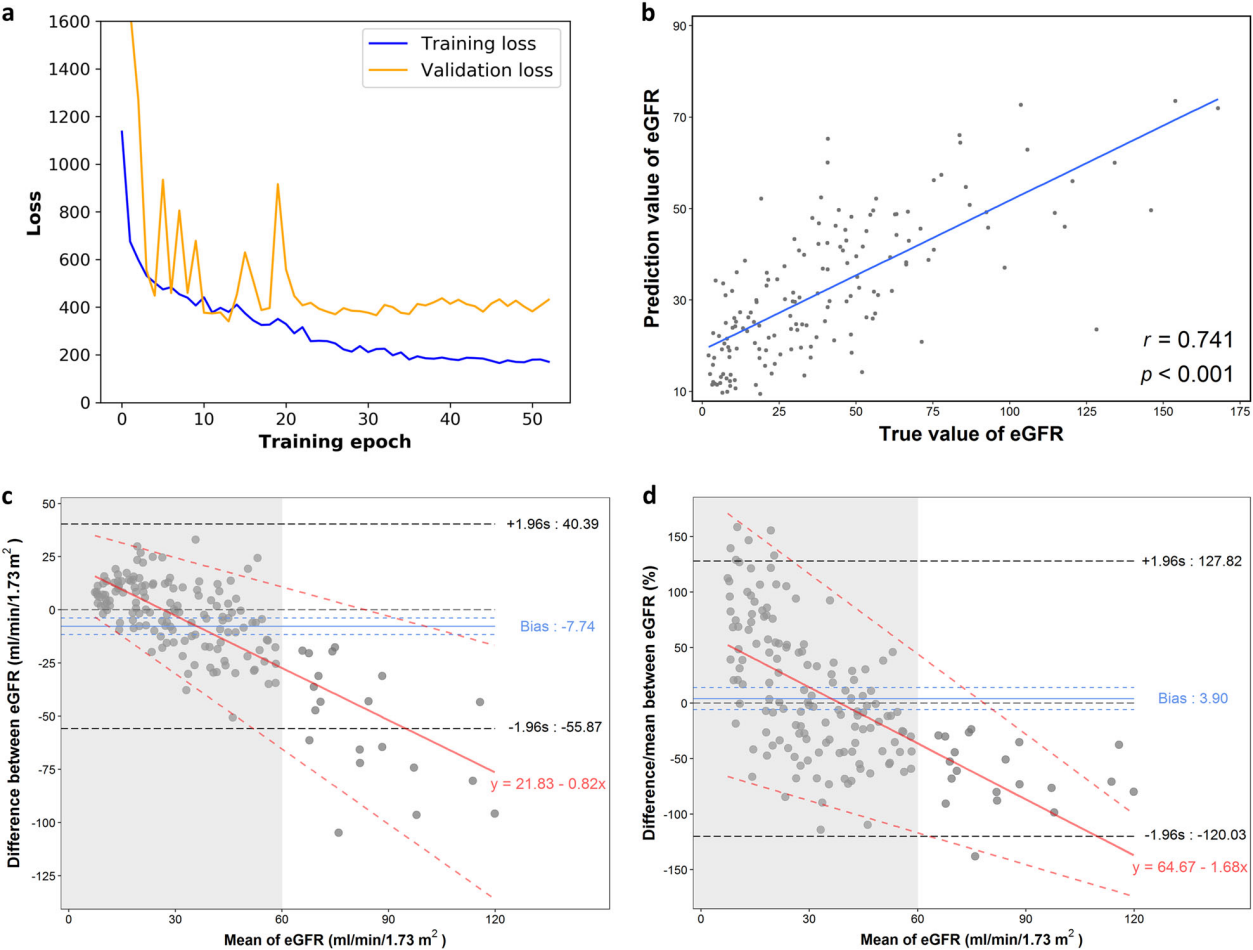
Performance of predicting continuous eGFR (estimated glomerular filtration rate) levels. **a** Learning curve of the ResNet model. Because we restored the model with minimum validation loss, in this case, we kept the model at epoch 14, where the smallest overfitting occurred. **b** Scatter plot of both predicted and actual eGFRs with a linear regression prediction line. γ, Pearson correlation coefficient. **c** Bland-Altman plot of difference between predicted and actual eGFR (predicted- actual eGFR) against mean eGFR. The blue solid line indicates the mean of difference from zero (thin black dotted line) with crude 95% confidence interval (blue dotted line) and the bold black dotted line represents the 95% crude limits of agreement. A linear regression line (red line) with 95% limits of agreement (red dotted line) characterizes the relationship between mean difference and the magnitude of eGFR with a slope of −0.82 (*p* value < 0.01). **d** Bland-Altman plot where mean differences are presented in percentage. Shaded grey areas in (**c**) and (**d**) represent the range of mean eGFR less than 60 ml/min/1.73 m^2^

The architecture and training process of the proposed convolutional neural networks (CNNs) are detailed in the Methods section and summarized in Figs 1 and 2. The learning curve of our ResNet model is shown in Fig. 3a. For predicting continuous eGFR, the aggregated model achieved a correlation of 0.741 and a mean absolute error (MAE) of 17.605 on the testing dataset after averaging the results from 10 models (Supplemental Table 1).

The relationship between predicted and actual eGFRs was visualized using a scatter plot (Fig. 3b). Despite achieving a satisfactory Pearson’s correlation coefficient of 0.74, the Bland-Altman plot showed that a significant mean difference (predicted – actual eGFR) was −7.74 ml/min/1.73 m^2^ (95% CI, −11.57 ~ −3.91) and this difference increased with the magnitude of eGFR with a slope of −0.82 (*p* value < 0.001) (Fig. 3c). However, there was a both clinically and statistically non-significant 3.9% mean percent difference (95% CI, −5.98 ~13.77) with a slope of −1.68 (*p*-value < 0.001) (Fig. 3d) and overall agreement was satisfactory (Fig. 3c, d). We initially attributed this observation to the limited capacity within the ResNet model because its final fully connected layer is linearly activated. However, even after calibration of the predicted eGFRs by using a high-degree polynomial regression model, the MAE reduced by only 0.1. Therefore, this issue cannot be simply explained by the model’s insufficient capacity. The root cause may be the uneven distribution of measured eGFRs in the selected dataset (Supplemental Fig. 1). More than 85% of sonographic studies with an eGFR of <60 ml/min/1.73 m^2^ had a learned regressor too conservative to spread the predictions.

### CKD classification through extreme gradient-boosting tree compared with that by nephrologists

**Fig. 4:**
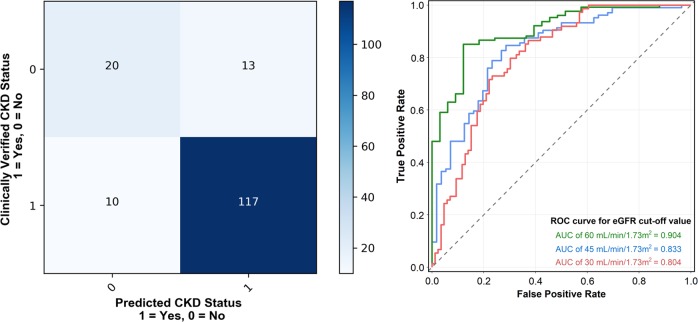
Performance of predicting CKD status. **a** Confusion matrix of the CKD status classification (eGFR < 60 ml/min/1.73 m^2^). **b** ROC curves of the CKD status classification using different eGFR cutoff values based on our proposed CNN model

For classifying eGFR with a threshold of 60 ml/min/1.73 m^2^, our model achieved an overall accuracy of 85.6% and area under receiver operating characteristic (ROC) curve (AUC) of 0.904. The classification performance is summarized in Supplemental Table 2 and Fig. 4. The attained specificity was up to 92.1%, demonstrating the effectiveness of our deep learning algorithm for assessing CKD by using ultrasound images (Supplemental Table 2). However, the sensitivity of our algorithm was only moderate (approximately 60.7%). The almost perfect agreement (B statistic, 0.81) between CNN-based eGFR and serum creatinine-based eGFR in the classification prediction of CKD was observed (Supplementary Table 2). When we set a stricter eGFR threshold (45 ml/min/1.73 m^2^), the results were similar with an overall accuracy of 78% and AUC of 0.83 (Fig. 4).

We evaluated how nephrologists performed on the testing data set compared with our proposed approach. We invited four nephrologists who had practiced nephrology for more than 10 years, with an annual volume of kidney sonography procedures larger than 800, to assess the images of the testing dataset. Our approach achieved average accuracy, precision, recall, and F1 score of 0.856, 0.913, 0.906, and 0.909, respectively, thus outperforming most nephrologists by a substantial margin. One nephrologist demonstrated the best precision (0.957) but the worst recall (0.528), demonstrating that they misclassified numerous samples to an eGFR of <60 ml/min/1.73 m^2^. According to these results, our proposed method reached nephrologist-level accuracy and provided reliable discrimination in practice. The results are summarized in Supplemental Table 3.

### Performance comparison with other CNN architectures and traditional machine learning approaches

To objectively demonstrate the feasibility and efficiency of the CNN architecture we chose, ResNet-101, we evaluated whether other state-of-the-art architectures such as Inception V4^22^ and VGG-19^23^ can improve the performance trade-offs considering MAE, correlation, Fused Multiply-Adds (FMA), and model size on eGFR prediction compared that of the reference ResNet-101. Despite VGG-19 did improve 3.1% of MAE, this model required 2.5-fold more computational operations and 3.2-fold larger model size compared that of ResNet-101 (Supplementary Table 4). We additionally compared the performance of common traditional machine learning approaches such as histogram of oriented gradients (HOG),^24^ local binary pattern (LBP),^25^ and Oriented FAST and Rotated BRIEF (ORB)^26^ with ResNet-101 from the perspective of CKD classification, none of them outperformed the ResNet-101 (Supplementary Table 5). Overall, specifically for the present task, ResNet-101 is the most cost-efficient considering the balance among model simplicity, model size, and performance.

## Discussion

Kidney sonography has long been a convenient point-of-care diagnostic tool in nephrology. With the advancements in deep CNNs, artificial intelligence (AI) can be introduced for real-time interpretation of kidney sonography—an essential first step toward a wide telemedicine outreach for effectively screening CKD in a community setting. We attempt to use a deep learning algorithm to predict eGFR and CKD status in a study population with various degrees of CKD (stages 1–5). The proposed algorithm moderately predicts continuous eGFR. Furthermore, it can reliably determine whether eGFR is below 60 ml/min/1.73 m^2^, with an accuracy superior to that of senior nephrologists. Kidney function is particularly prone to irreversible decline after eGFR becomes <60 ml/min/1.73 m^2^. Thus, this algorithm is applicable because it helps optimize cost-effective CKD screening practices without laboratory testing, particularly in settings with limited health care resources. Notably, this algorithm provides a real-time diagnosis and patient referral. The present study also demonstrates the possible role of AI in turning conventional images into functional screening and diagnostic tools – this type of automation will be pervasive in the era of AI and Big Data. For instance, prior studies have applied AI in automatically identifying glomeruli to standardize renal biopsy interpretation,^27–29^ and even trying to predict kidney function.^30^ While the predictive accuracy for eGFR or CKD is not perfectly satisfied with current clinical practice at this stage, our proposed deep learning algorithm is to complement existing screening or case-finding instruments, rather than to replace them.

Surging CKD-related health care costs burden both developed and developing economies.^31^ In the United States, CKD prevalence is expected to increase by 16.7% by 2030. A study showed that adults aged 30–49 years without CKD at baseline had a residual lifetime incidence of CKD as high as 54%.^32^ Global trends in population aging may increase CKD prevalence because aging is a pertinent risk factor for CKD.^33^ The primary prevention of CKD through early detection is recommended particularly among high-risk patients with diabetes and hypertension.^34^ However, the screening relies on serum creatinine (invasive) and urine protein (noninvasive) level measurement, require blood and urine specimens to be analyzed by laboratory personnel by using laboratory equipment of appropriate quality, respectively. The mass screening for CKD in the general population by measuring serum creatinine levels is expensive in most health care systems because of the costs and invasiveness involved. Proteinuria-based screening, such as routine urinalysis, is more acceptable by the general population because it is noninvasive. Among the 10 studies enrolled in a recent systematic review of the cost-effectiveness of primary CKD screening, 8 used proteinuria-based screening, such as urine dipstick testing and protein-to-creatinine ratio measurement, reflecting the well perceived patient acceptance of noninvasive urine-based tests.^34^ However, the poor screening performance of routine urinalysis, with 93% specificity but only 11% sensitivity, in detecting early CKD among the general population reveals the need for new screening methods.^35^

The cost-effectiveness of mass screening in general population for CKD has long been debated.^36,37^ For instance, the American College of Physicians’ 2013 clinical practice guideline for managing stage 1–3 CKD recommends against universal CKD screening among asymptomatic adults because evidence from randomized trials supporting the benefits of regularly screening for CKD is insufficient.^38^ By contrast, the American Society of Nephrology strongly recommends regular screening of CKD, given its clinical silence and preventable progression with relatively low cost of testing.^39,40^ More conclusive research is required to fill the practice gaps in key areas ranging from the identification of novel and cost-effective techniques to the development of systemic evaluation methods that are economically efficient for mass CKD screening.

Over the past decade, Taiwan has demonstrated the highest end-stage renal disease (ESRD) incidence and prevalence worldwide, and they are still increasing, despite the considerable amount of resources available for CKD care programs.^41^ Therefore, cost-effective universal screening for CKD may aid Taiwan. On the basis of our current study, we propose a two-stage CKD screening approach: Stage 1 comprises kidney ultrasound image screening using our AI-aided screening method, whereas stage 2 comprises serum creatinine quantification for identifying missed true positives. This two-stage screening model offers advantage in terms of logistics supportability (wide availability of ultrasound machines and pervasive Internet services in Taiwan) and sustainment with financial capability and affordability. This AI-aided model also provides a potential complementary care model to routine urinalysis or serum creatinine measurement for primary CKD screening. Conducting a comprehensive economic analysis to examine the cost-effectiveness of our proposed AI-aided screening model is beyond the scope of this study. Future studies should examine the economic viability of our model. Furthermore, our approach should be extended to mobile applications to augment its impact on health care efficiency and quality.

Ensuring a sufficient number of samples is a prerequisite for training a robust deep learning model. We evaluated how much performance improvement can be achieved by increasing the data size through the following steps of the experimental study: (1) Use a sample 10% of the entire dataset without replacing the experimental data set. (2) Train several ResNet models by using the experimental dataset under different random seeds, and average their testing performance to obtain a robust evaluation. (3) Randomly add additional 10% of the entire dataset to the experimental dataset. (4) Repeat Steps 2 and 3 until all data are added to the experimental dataset. We did not apply bootstrap aggregation in this experiment. The results are shown in Supplemental Fig. 2a: a clear declining trend in testing loss was noted when data size increased. Simultaneously, Pearson’s correlation coefficient improved (Supplemental Fig. 2a). For instance, compared with 10% of the entire dataset, the testing loss using 50% of the entire dataset resulted in a twofold increase in performance and increase in correlation coefficient from 0.53 to 0.66. Therefore, the testing performance of our model may improve when more sonographic studies are available.^42^

Relatively few sonographic studies in our dataset (15%) reported a normal eGFR of greater than 60 ml/min/1.73 m^2^. To resolve this data imbalance for CKD status classification, we reduced the weight of the samples using eGFR of <60 ml/min/1.73 m^2^ by a factor of 0.25 to balance their effects, classify the loss, and summarize the predictive performance based on unscaled data (Supplemental Table 6). The overall accuracy was comparable to the results based on scaled data, despite the inherent tradeoff between sensitivity and specificity. Both approaches can adequately be the first screening test in our proposed two-stage mass screening model. Because CKD and ESRD are highly prevalent in Taiwan, the best positive predictive power can be obtained by adjusting the algorithm targeting high specificity. This sensitivity experiment indicated the strategic flexibility of our deep neural network algorithm.

Numerous possibilities exist for functional AI-powered automation to support the efficiency of health care. Our proposed deep learning algorithm offers the possibility of noninvasive assessment of kidney function and represents a fundamental step for realizing the potential of transforming kidney ultrasound into an effective, real-time screening tool. With a diagnostic accuracy comparable to the predictions of experienced nephrologists, our CNN model has the potential to improve the cost efficiency of universal CKD screening, for instance, by selecting high-risk patients using kidney ultrasound in the first round of a two-stage screening model.

## Methods

### Clinical information

Taiwan’s National Health Insurance launched the Integrated Care of CKD project in 2002. China Medical University Hospital (CMUH), a tertiary medical center in Central Taiwan, joined this program in 2003, prospectively enrolling consecutive patients with CKD willing to participate.^41^ CKD diagnosis was based on the criteria of the National Kidney Foundation’s Kidney Disease Outcomes Quality Initiative’s Clinical Practice Guidelines for CKD.^41,43^ The patients in this program were regularly followed at the outpatient department; they routinely underwent at least one kidney sonographic study. In Taiwan, almost all kidney sonographic studies are performed and interpreted by nephrologists. Biochemical markers of renal injury, including serum creatinine and blood urea nitrogen levels as well as spot urine protein-to-creatinine ratio, were measured at least every 12 weeks or more frequently. Since 2003, CMUH has implemented electronic medical records (EMRs) for care management; therefore, we integrated the data of CMUH’s pre-ESRD program with CMUH’s EMRs containing laboratory test results, medications, special procedures, medical images, and admission records.^44^ We initially enrolled 8,281 CMUH pre-ESRD patients aged 20–89 years, with a total of 203,353 sonographic images; their eGFR was measured within 4 weeks before or after the day of the kidney sonography. The study was approved with waived informed consent by the Research Ethical Committee/Institutional Review Board of the China Medical University Hospital in Taiwan (Approval no.: CMUH105-REC3–068 and CMUH106-REC3–118).

The eGFR was estimated using the abbreviated MDRD equation (eGFR = 186 × creatinine^−1.154^ × age^−0.203^ × 1.212 [if black] × 0.742 [if female]).^45^ The serum creatinine level closest to and within the 4 weeks before and after the day of the kidney sonography was used to define the labeled eGFR. The sociodemographic variables collected during the enrollment interview were age, sex, education, cigarette smoking status, and alcohol consumption. Diabetes mellitus and hypertension were defined by the physicians’ clinical diagnoses based on the patients’ International Classification of Diseases codes and glucose-lowering or blood pressure-lowering agent use. History of cardiovascular disease was defined as documented coronary artery disease, myocardial infarction and stroke in the EMRs.

### Data information

All kidney ultrasound studies were performed by board-certified nephrologists and deidentified with waived consent, complying with the Institutional Review Board of CMUH. We selected studies performed after 2014 that used GE ultrasound systems (LOGIQ E9 and LOGIQ P3, GE Healthcare, Milwaukee, WI, USA) for higher image quality, with regard to sharpness, contrast, and noise, compared with images from prior systems (before 2014). The original Digital Imaging and Communications in Medicine files were then converted into Portable Network Graphics images; 37,696 images were selected from the two GE models with two different sizes: 960 × 720 for LOGIQ E9 and 820 × 614 for LOGIQ P3.

In general, nephrologists determine an individual’s kidney length by obtaining images of the best possible quality to capture the maximum observable kidney length. We then used the template matching technique to detect the presence of this specific annotation pattern in every image and then filter out those images without length annotations measuring kidney size. We selected these high-quality images to train our deep learning model, with the final dataset containing 1,446 uniquely identifiable primary sonographic studies of 1299 patients. Each sonographic study provided at least one image each of the right and the left kidneys. Each sonographic study also served as the primary unique input, with the final database comprising 4,505 images. The selection flow chart is presented in Supplemental Fig. 3. For the selected 4,505 images, we applied the “findContours” function of the cv2 module in Python to isolate “bean-shaped” kidneys from irrelevant information surrounding the kidneys, such as the supplier’s logo, which may have obfuscated the learning accuracy of our proposed CNN.

### Model Selection: prediction of continuous eGFRs through CNNs

We predicted eGFRs based on patients’ kidney ultrasound images by using deep CNNs. Our neural network architecture, as illustrated in Fig. 1a, was referenced from the ResNet-101 model.^46^ In brief, the ResNet-101 model comprises a bunch of residual blocks, with each block being a combination of convolution and identity-mapping layers, resulting in a total of 101 layers (Fig. 1b). To predict the patients’ eGFRs, we replaced the last 1000-class classifier in the ResNet-101 model by using a regressor of consecutive fully connected layers, comprising 512 (FC1), 512 (FC2), 256 (FC3), and 1 (output), as illustrated in Fig. 1a. We employed a dropout layer to reduce overfitting between every two consecutive fully connected layers, where the dropout probability was determined using the grid search method.^47^ The activation function of all layers except the output layer used rectified linear units; the output layer adopted a linear activation function because this prediction task was a regression-type problem with the output values ranging from 0 to >100. For this regression-type prediction problem, we optimized the mean squared error defined as follows:

$${\mathrm{MSE}} = \frac{1}{n}\mathop {\sum }\limits_{i = 1}^n \left( {\hat Y_i - Y_i} \right)^2,$$

where $\hat Y_i$ and *Y*_*t*_ are the predicted and actual eGFRs of sample *i*, respectively.

Motivated by the observation^44^ that the earlier features of a convolution network are generally not specific to a particular task and thus transferable to other tasks, we explored different combinations of freezing residual blocks and found that by keeping the first residual block fixed, a minimal mean squared error was achieved over the validation dataset (Supplemental Table 7).^48^ By contrast, the parameters of the regressor were randomly initialized using a Gaussian distribution, with a mean of zero and standard deviation of 0.1. Except the regressor, we considered ResNet-101 pretrained on ImageNet as an initialization for the rest of our network weights.

### Model selection: prediction of irreversible CKD status through extreme gradient-boosting tree

Clinically, an eGFR of <60 ml/min/1.73 m^2^ denotes prognostic significance of reduced kidney function. At this stage, patients must receive multidisciplinary nephrological care.^49^ To evaluate whether our CNN model accurately detects an irreversible CKD status, we reformulated the original regression problem to a binary classification problem by predicting whether a patient’s eGFR was lower than the cutoff threshold of 60 ml/min/1.73 m^2^.

Here we treated the ResNet model that was well-trained in Section 3 as a fixed feature extractor and computed a 256-dimension vector for every image containing the activation of the last fully connected layer (FC3) of the ResNet model in Section 3. We demoted these 256-dimension features as image codes. After extracting these codes from the images, we trained an eXtreme Gradient-Boosting model (XGBoost), a scalable end-to-end tree boosting model proposed by Chen and Guestrin,^50^ to identify whether the corresponding eGFR value was below the 60-ml/min/1.73 m^2^ threshold. The objective function of this binary classification problem was of minimizing binary entropy loss; the hyperparameters of our XGBoost model were determined using the grid search method.^47^ For the implementation of XGBoost in Python, the finalized hyperparameters were set as tree depth = 3, learning rate = 0.1, data subsampling = 50%, column sampling = 50%, and scale the positive sample weight by 0.25; the remaining components were set using the default setting. The XGBoost model output a probability of eGFR below the cutoff threshold (60 ml/min/1.73 m^2^).

### Training phase

All sonographic studies were partitioned into nontesting (90%) and testing (10%) groups based on the unique and hashed patient identification key to ensure mutually exclusive flow of patients into different groups. The sample size planning was based on the MAE learning curves (Supplementary Fig. 2a). We used all images from sonographic studies in the same group as the dataset of that group. The testing dataset was not employed in this phase. We adopted the bootstrap aggregation (also called bagging) technique, a model ensemble algorithm, to improve the stability and accuracy of our deep learning model. During bagging, we uniformly sampled from the nontesting dataset with replacement to assemble a double sized dataset as the training dataset. Some duplicate sonographic studies existed in a training dataset, and it is expected to have a fraction of 86.4% of unique sonographic studies of the entire nontesting dataset.^51^ We considered such sonographic studies from a training dataset as the validation (out-of-bag) dataset. We repeated the above sampling process 10 times to obtain 10 pairs of the training and out-of-bag datasets. For each pair, we trained a ResNet model and XGBoost for eGFR prediction and CKD status classification, respectively. In the final evaluation (testing) phase, we averaged the output of 10 independent models from bagging as the final prediction. The flowchart is presented in Fig. 2.

**Fig. 5:**
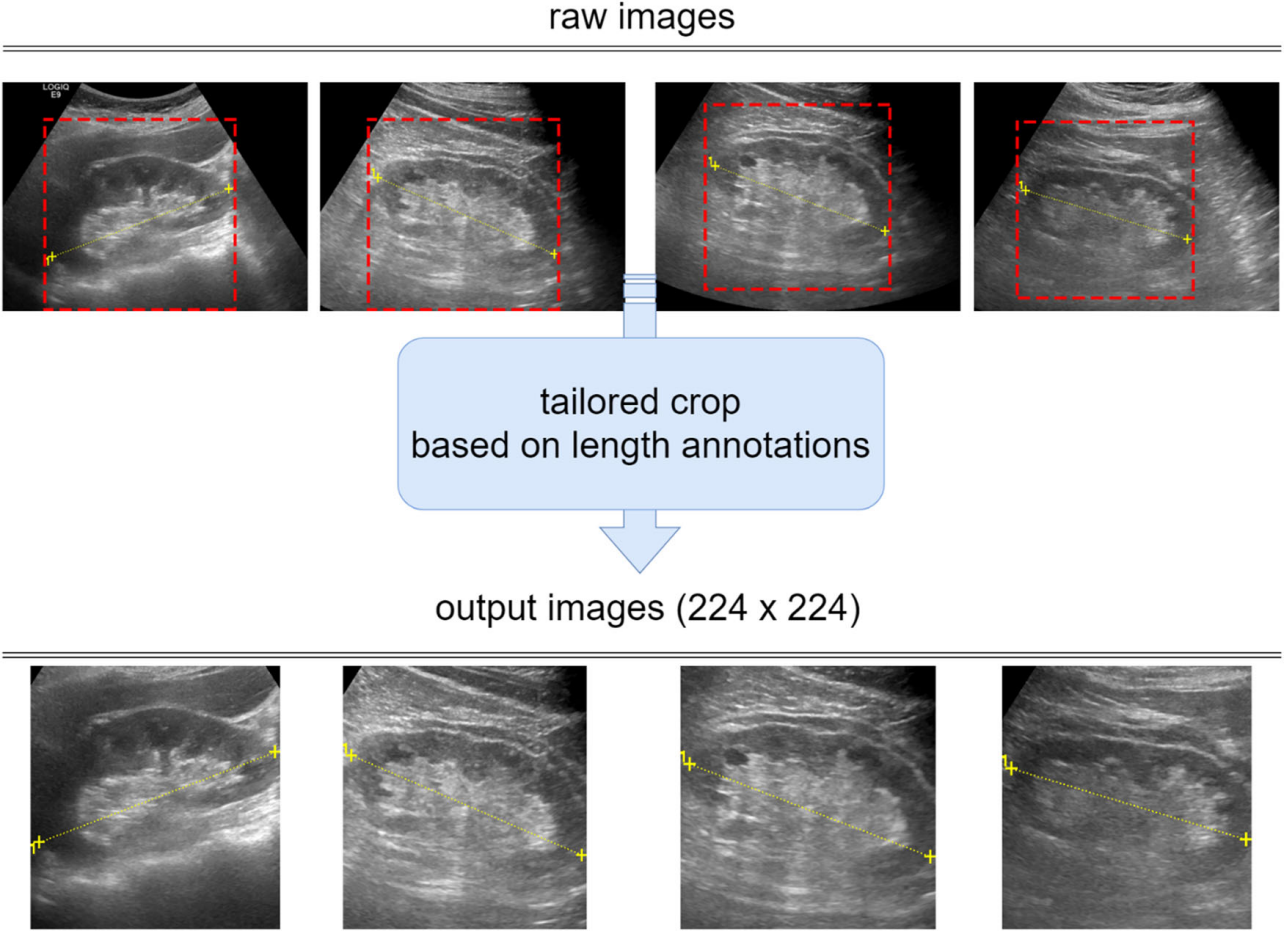
Tailored image-cropping method, based on two markers that annotated the kidney length, was used to remove the irrelevant peripheral region of the kidneys. To unify the image size to our neural network model, we resized cropped images to 224 × 224 pixels. Data augmentation schemes comprising shift, rotation, and horizontal flip were performed

Before feeding images into our ResNet model, we conducted a tailored image-cropping method, based on two markers annotating the kidney length, to remove the irrelevant peripheral region of the kidneys. We first identified the positions of the two markers $(x_1,y_1)$ and $(x_2,y_2)$ and calculated their distance and middle point, denoted as *d* and $(x_c,y_c)$, respectively. Next, we cropped the square region centered at $(x_c,y_c)$ with a length *d*. To unify the size of the input images, we resized the cropped images to 224 × 224 pixels and normalized each pixel value based on the mean and standard deviation of the images in the ImageNet dataset. During training, three image augmentation schemes—namely shift along *x* and *y* axes (±10%), rotation (±40 degree), and horizontal flip—were applied independently, with each scheme having an 80% probability of occurrence. Several input images are presented in Fig. 5.

In Section 3, our ResNet model was trained using Adam optimizer, which automatically adapted the learning rate for every parameter and considered the momentum of gradients during optimization using a batch size of 128 at a time for gradient calculation.^52^ An initial learning rate of 10^−4^ was used, which was then reduced by a factor of 10 after validation loss plateaued over 10 epochs. We imposed an L2 regularization of 10^−5^ on the network parameters (also called weight decay) to achieve better model generalization. We adopted an early stopping mechanism with a patience of 20 to prevent overfitting and retain the model at the minimum validation loss. We then aggregated the 10 ResNet models in the bagging process by averaging their predictions when evaluating the dataset testing. In Section 4, we trained a corresponding XGBoost model to identify whether a patient’s eGFR was <60 ml/min/1.73 m^2^ by using the codes extracted from the ResNet model as inputs. The nontesting and testing members were the same as in Section 3. Finally, we obtained 10 XGBoost models for predicting an irreversible CKD status and then restored these models for the next testing phase.

### Evaluation(testing) phase

For each sonographic study in the testing group, we selected the ultrasound kidney image with the longest annotated length for the final testing dataset. No image augmentation was performed in the evaluation phase. To reduce the variance among the models, we averaged the outputs from the 10 ResNet models, restored in the bagging process as the final eGFR prediction. We quantified the prediction results by using the following metrics: MAE, Pearson’s correlation, and R-squared.

$\hat Y_i = \frac{1}{{10}}\mathop {\sum }\nolimits_{j = 1}^{10} y_{ij}$, where *y*_*ij*_ is the prediction of input sample *i* by the ResNet-*j* model

${\mathrm{MAE}} = \frac{1}{n}\mathop {\sum }\nolimits_{i = 1}^n \left| {\hat Y_i - Y_i} \right|$, where *Y*_*i*_ is the measured eGFR value of input sample *i*

$$\rho _{Y,\hat Y} = \frac{{cov(Y,\hat Y)}}{{\sigma _Y \cdot \sigma _{\hat Y}}}$$

$$R^2 = 1 - \frac{{SS_{res}}}{{SS_{tot}}}$$, where $$SS_{tot} = \mathop {\sum }\limits_{i = 1}^n (Y_i - \overline {Y_i} )^2$$ and $$SS_{res} = \mathop {\sum }\limits_{i = 1}^n (\hat Y_i - \overline {Y_i} )^2.$$

For evaluating the testing performance for classifying CKD status, we averaged the output probabilities from 10 restored XGBoost models used in the previous training phase. The classification probability threshold was set to 0.5 as follows:

- $\hat P_i = \frac{1}{{10}}\mathop {\sum }\nolimits_{j = 1}^{10} P_{ij}$, where *P*_*ij*_ is the prediction of input sample *i* by the XGBoost-*j* model
- $$\hat Y_i = \left\{ {\begin{array}{*{20}{c}} {0,\hat P_i \,<\, threshold} \\ {1,\hat P_i \ge threshold} \end{array}} \right.$$, where $threshold$ is set at 0.5.

True positive rate (TPR), true negative rate (TNR), false positive rate (FPR), and false negative rate (FNR) were used for calculating accuracy, precision, recall, F1 score and plotting the Receiver Operating Characteristic (ROC) curve and the estimated Area Under Curve (AUC). We also evaluate the agreement between CNN-based eGFR and serum creatinine-based eGFR in the classification of CKD by B-statistic due to the highly symmetrically imbalanced nature of the present data. The definitions of TRP, FPR, TNR and FNR are provided in the Supplementary Text. To examine the model’s reliability, we leveraged the bootstrap method to construct 95% bootstrap confidence intervals, evaluating the model’s performance on 10,000 bootstrap testing datasets, sampled from the testing dataset with replacement. We regarded the 2.5th and 97.5th percentiles of the evaluation results as the 95% bootstrap confidence intervals. The bootstrap confidence intervals of the accuracy, precision, recall, and F1 score are presented in the Results section.

## Supplementary information

Supplementary Material

## Data Availability

The data that support the findings of this study are available on request from the corresponding author, CCK. The data are not publicly available due to them containing information that could compromise research participant privacy.

## Supplementary information

**Supplementary Information** accompanies the paper on the *npj Digital Medicine* website (10.1038/s41746-019-0104-2).

## Code availability

The full code developed in this study is available from chinchik@gmail.com on reasonable request.
