# Supplementary material for: Automation of the kidney function prediction and classification through ultrasound-based kidney imaging using deep learning
Source: NPJ Digit Med. 2019 Apr 26;2:29. doi: 10.1038/s41746-019-0104-2 (PMC6550224; doi:10.1038/s41746-019-0104-2)
Supplement: Supplementary file 1 — Supplementary Material [file 41746_2019_104_MOESM1_ESM.pdf]

Supplementary Text. Definitions of true positive rate (TPR), true negative rate (TNR), false positive rate (FPR), and false negative rate (FNR) and formulas of precision, recall, accuracy, and F1 score that are calculated by Python "sklearn" package.

- **True positive rate (TPR):** the ratio between the number of positive events correctly categorized as positive (TP) and the total number of actual positive events
- **True negative rate (TNR):** the ratio between the number of negative events correctly categorized as negative (TN) and the total number of actual negative events
- **False positive rate (FPR):** the ratio between the number of negative events wrongly categorized as positive (FP) and the total number of actual negative events
- **False negative rate (FNR):** the ratio between the number of positive events wrongly categorized as negative (FN) and the total number of actual positive events
- **Precision** $= \frac{TP}{TP+FP}$
- **Recall** $= \frac{TP}{TP+FN}$
- **Accuracy** $= \frac{TP+TN}{TP+TN+FP+FN}$
- **F1 score** $= 2 \times \left(\frac{\text{Precision}\times\text{Recall}}{\text{Precision}+\text{Recall}}\right)$

Supplemental Fig. 1. Violin plots show the distribution of eGFR labeled for selected kidney sonographic images by patient's characteristics including whether being in the testing dataset, elderly, male, diabetic and hypertensive. Box plots showing the median and interquartile range of eGFR in each subgroup are embedded in each violin plot.

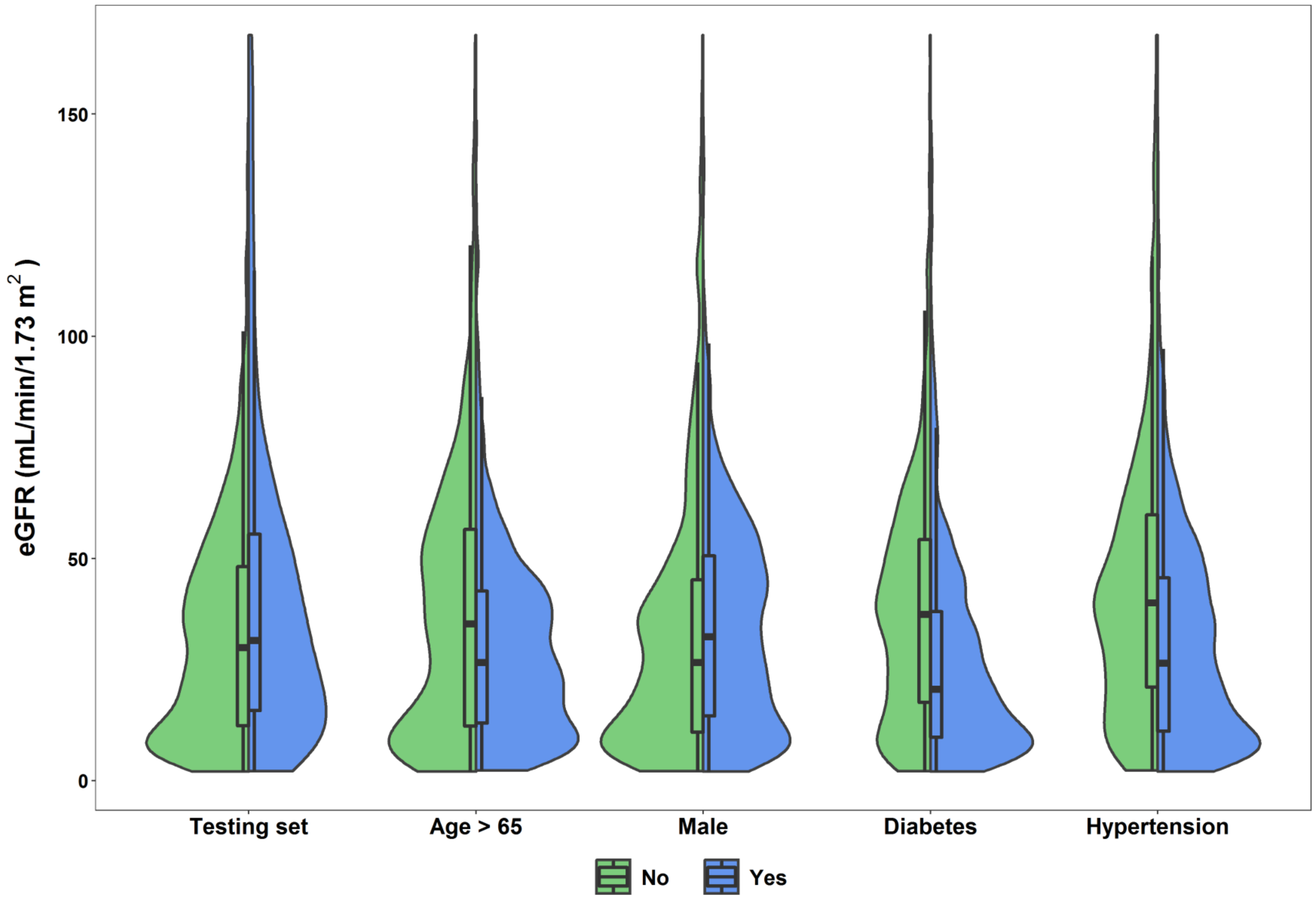

Supplemental Fig. 2. The testing performance versus the percentage of the entire training dataset. **(a)** MAE (blue) and correlation coefficient (red) both improves from increasing data size. **(b)** The performance of CKD status classification without scaling the weight of above-60 samples. CKD, chronic kidney disease; MAE, mean absolute error.

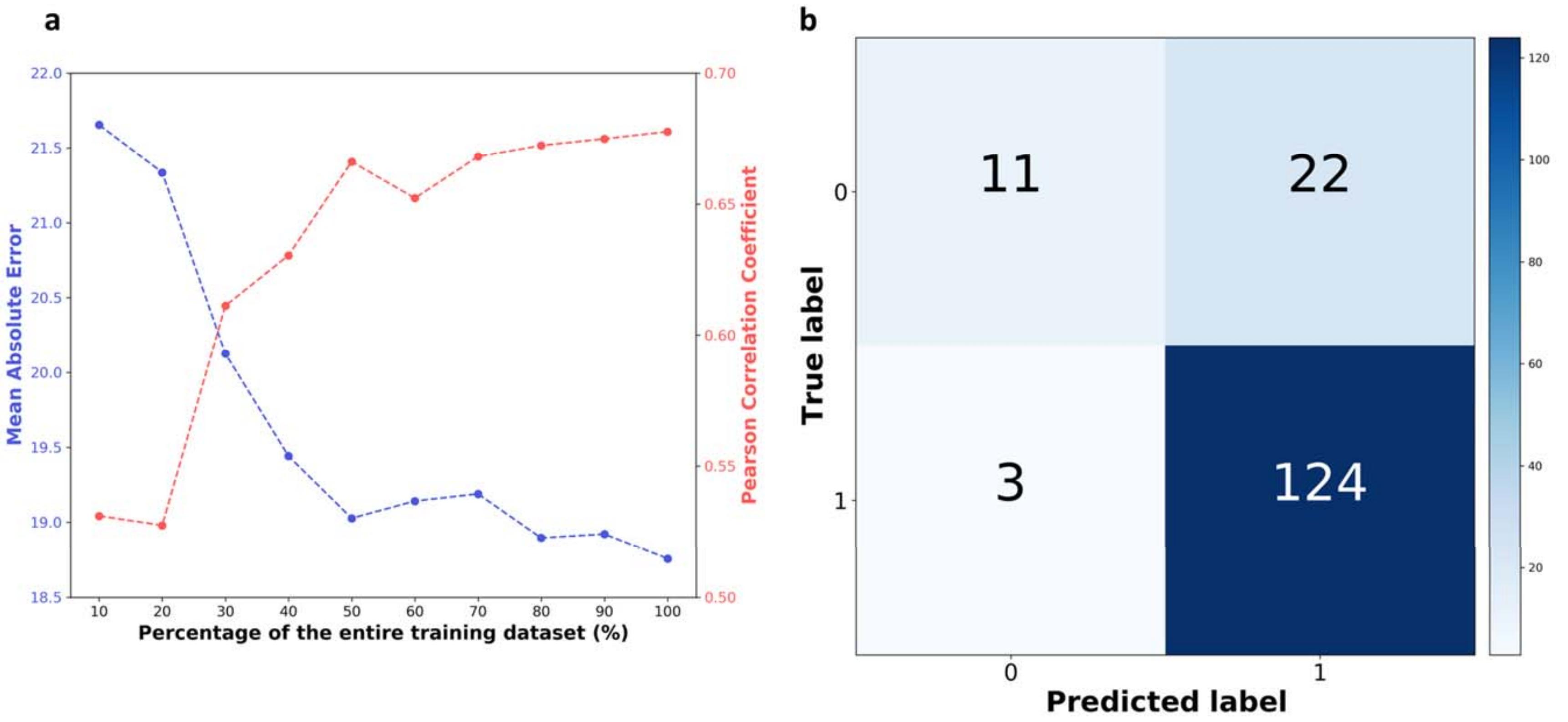

Supplemental Fig. 3. Flow diagram of image selection process. **Abbreviations:** eGFR, estimated glomerular filtration rate; CMUH, China Medical University Hospital. (*) indicates cases with severe hydronephrosis, severe polycystic kidney disease, and kidney cancers that significantly compromise the kidney parenchyma.

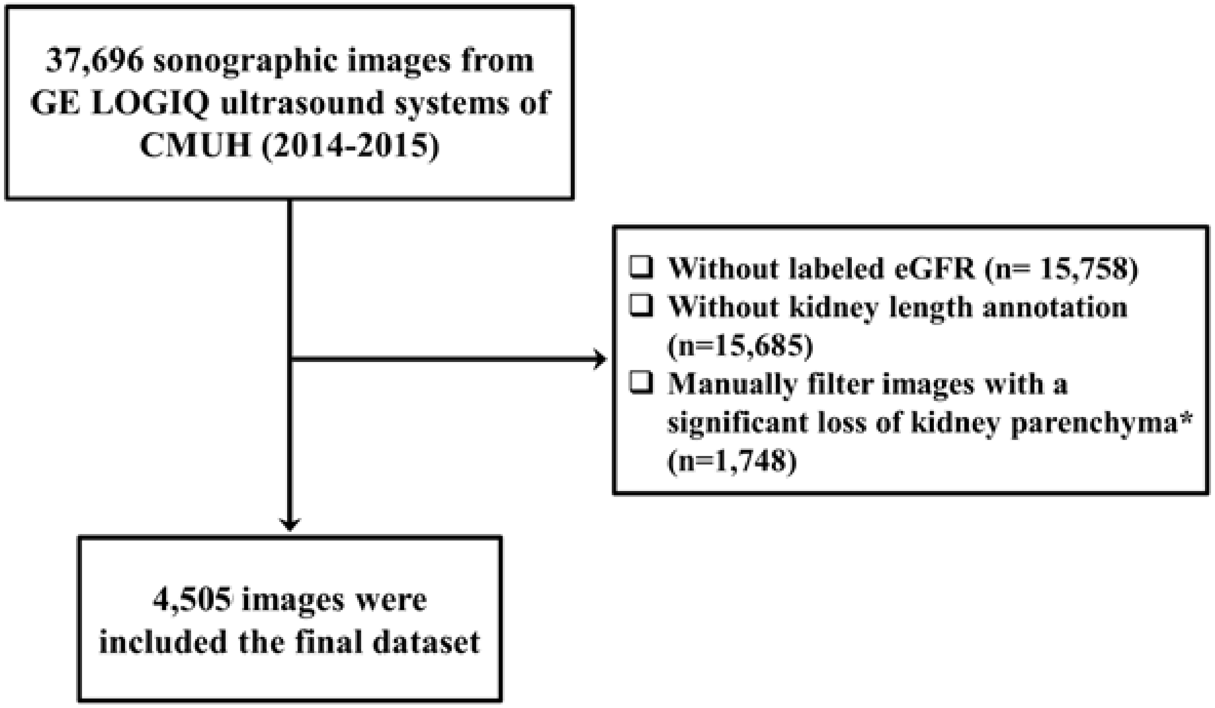

Supplemental Table 1. The performance of predicting continuous eGFR (estimated glomerular filtration rate) by ensembling the 10 models.

	MAE	Correlation	R-squared
**10-model ensemble**	17.605	0.741	0.421

Supplemental Table 2. Comparison of performance in determining CKD status based on different eGFR (estimated glomerular filtration rate) cut-off values. AUC, area under curve; FN, false negative; FP, false positive; TN, true negative; TP, true positive.

Cutoff value	30 ml/min/1.73m^2		45 ml/min/1.73m^2		60 ml/min/1.73m^2	
**Confusion matrix**	TN = 66	FP = 20	TN = 41	FP = 15	TN = 20	FP = 13
	FN = 21	TP = 53	FN = 20	TP = 84	FN = 10	TP = 117
**Accuracy**	0.7438		0.7813		0.8563	
**AUC**	0.8036		0.8326		0.9036	
**Kappa statistic**	0.4831		0.5289		0.5458	
**B-statistic**	0.5572		0.6372		0.8051	
**Sensitivity**	0.7027		0.8077		0.9213	
**Specificity**	0.7791		0.7321		0.6061	

Supplemental Table 3. Performance comparison among the proposed convolutional neural network (CNN) and four veteran nephrologists evaluated by bootstrap confidence interval. Numbers in bold font indicate the highest performance in the column (e.g., accuracy).

	Accuracy (95% CI)	Precision (95% CI)	Recall (95% CI)	F1 score (95% CI)
**Nephrologist 1**	0.801 (0.753, 0.848)	0.893 (0.851, 0.935)	0.852 (0.805, 0.899)	0.871 (0.836, 0.904)
**Nephrologist 2**	0.763 (0.713, 0.814)	0.881 (0.838, 0.929)	0.811 (0.761, 0.864)	0.844 (0.806, 0.881)
**Nephrologist 3**	0.650 (0.593, 0.707)	0.917 (0.875, 0.963)	0.614 (0.549, 0.679)	0.735 (0.683, 0.785)
**Nephrologist 4**	0.607 (0.549, 0.667)	**0.957 (0.927, 1.000)**	0.528 (0.461, 0.595)	0.680 (0.619, 0.737)
**Proposed CNN**	**0.856 (0.816, 0.898)**	0.913 (0.877, 0.951)	**0.906 (0.868, 0.947)**	**0.909 (0.881, 0.937)**

Supplemental Table 4. Comparison of the performance of predicting continuous eGFR (estimated glomerular filtration rate) by ensembling 10 models based on different CNN architectures – ResNet-101, Inception V4 and VGG-19. *Note that FMA is the abbreviation of "Fused Multiply-Adds", and we used this measure as a rough approximation of computational cost as these architectures were applied.[1]

	MAE	Correlation	R-squared	Model Size (MB)	FMA*
**ResNet-101**	17.605	0.741	0.421	172	**7.8×10^9**
**Inception V4**	17.532	0.738	0.471	157	12.3×10^9
**VGG-19**	17.060	0.753	0.489	550	19.7×10^9

Supplemental Table 5. Comparison of three conventional machine learning approaches (HOG, LBP, and ORB) with our proposed ResNet-101 model in terms of the predictive performance of CKD classification. HOG, histogram of oriented gradients; LBP, local binary pattern; ORB, Oriented FAST and Rotated BRIEF; AUC, area under curve; FN, false negative; FP, false positive; TN, true negative; TP, true positive.

Features	HOG	LBP	ORB	ResNet-101
**Dimension**	15876	256	12800	256
**Confusion matrix**	TN=9, FP=24<br>FN=6, TP=121	TN=3, FP=30<br>FN=12, TP=115	TN=2, FP=31<br>FN=7, TP=120	TN=20, FP=13<br>FN=10, TP=117
**Accuracy**	0.8125	0.7375	0.7625	**0.8563**
**AUC**	0.7182	0.5321	0.5101	**0.9036**

Supplemental Table 6. Performance comparison between two approaches: positive samples with or without scaled weights.

Positive weight	True Positive Rate	Ture Negative Rate	False Positive Rate	False Negative Rate	Accuracy	Area Under Curve
**with scaled**	117/127=92.1%	20/33=60.6%	13/33=39.4%	10/127=7.9%	85.6%	0.903
**without scaled**	124/127=97.6%	11/33=33.3%	22/33=66.7%	3/127=2.4%	84.3%	0.884

Supplemental Table 7. The performance comparison among different combination of fixed blocks in our ResNet model. MAE, mean absolute error.

Fixed blocks	MAE	Correlation	R-squared
**None**	18.337	0.605	0.367
**First**	17.655	0.636	0.406
**First and second**	17.709	0.630	0.398

References:

1. Howard, A.G., *et al.* MobileNets: Efficient Convolutional Neural Networks for Mobile Vision Applications. in *arXiv e-prints* (2017).
